# Reactive gaseous mercury is generated from chloralkali factories resulting in extreme concentrations of mercury in hair of workers

**DOI:** 10.1038/s41598-018-20544-5

**Published:** 2018-02-27

**Authors:** Abdelkarem A. S. Elgazali, Zuzana Gajdosechova, Zaigham Abbas, Enzo Lombi, Kirk G. Scheckel, Erica Donner, Heidelore Fiedler, Jörg Feldmann, Eva M. Krupp

**Affiliations:** 1 Trace Element Speciation Laboratory, University of Aberdeen, Department of Chemistry, Aberdeen, UK; 2 Government of Pakistan Ministry of Climate Change, LG & RD Complex, Islamabad, Pakistan; 3 Future Industries Institute, University of South Australia, Building X, Mawson Lakes Campus, Mawson Lakes, South Australia Australia; 4 United States Environmental Protection Agency, National Risk Management Research Laboratory, Cincinnati, OH 45224 USA; 5 United Nations Environmental Programme, Chemicals Branch, DTIE, 11-13 Chemin des Anemones, CH-1219 Chatelaine, Switzerland; 6 Present Address: Örebro University, School of Science and Technology, MTM Research Centre, SE-701 82 Örebro, Sweden

## Abstract

Occupational exposure of chloralkali workers to highly concentrated mercury (Hg) vapour has been linked to an increased risk of renal dysfunction and behavioural changes. It is generally believed that these workers are exposed to elemental Hg, which is used in abundance during the production process however, the lack in analytical techniques that would allow for identification of gaseous Hg species poses a challenge, which needs to be addressed in order to reach a consensus. Here, we present the results from simulated exposure studies, which provide sound evidence of higher adsorption rate of HgCl_2_ than Hg^0^ and its irreversible bonding on the surface of hair. We found that chloralkali workers were exposed to HgCl_2_, which accumulated in extremely high concentrations on the hair surface, more than 1,000 times higher than expected from unexposed subjects and was positively correlated with Hg levels in the finger- and toenails.

## Introduction

Atmospheric mercury (Hg) is usually categorized according to its physical and chemical properties into elemental Hg (Hg^0^), reactive gaseous or oxidized Hg (RGM) and particulate Hg (p-Hg). However, the generally crude definition of RGM is ill-founded and reflects the lack of analytical methodologies as it includes a large variety of gaseous inorganic Hg (InHg) species such as Hg(OH)_2_ and Hg-halide complexes^1^. Under typical conditions, Hg^0^ is by far the most abundant Hg species found in the atmosphere with only a small fraction present as RGM and p-Hg^2^. Nevertheless, air can be enriched with any p-Hg in the vicinity of emission sources or areas of high atmospheric concentration of gaseous oxidants such as Polar Regions^3,4^.

Chloralkali plants using Hg cell technology emitted globally over 28 t of Hg in 2010 exposing the surrounding environment and workers to high Hg pollution^5^. During the production of chlorine (Cl_2_), hydrogen (H_2_) and caustic soda (NaOH) via the electrolysis of a brine solution (NaCl), a thin layer of Hg^0^ is used as flowing cathode to dissociate the brine solution into chlorine and sodium typically at about 66 °C^6^. Although, only Hg^0^ should be present in the reaction cells, the high temperature process results in the saturation of the cell and plant air with Hg^0^ and Cl_2_, thus creating favorable conditions for the production of HgCl_2_. Yet, not only there is a significant lack of data, which could provide good estimate of the amount of RGM being released, but the identity of Hg species formed with chloralkali plants is also very rarely addressed. The most commonly employed methods, which are using either selective adsorption in tubular denuders^7^ and ion exchange membranes^8^, or a selective removal from the gas stream by scrubbing solution in the refluxing mist chamber^1^ are limited to the distinction between Hg^0^ and other RGM. Additionally, while previous studies showed that the concentration of the latter accounts only for up to 4% of total Hg emitted from the chloralkali roof vents under normal operation condition, it was suggested that its concentration can significantly increase during the invasive maintenance procedure^9,10^.

Despite their generally low ambient air concentration, RGM species are of a particular concern due to their high water solubility 1.4 × 10^6^ M atm^−1^ at 25 °C (0.11 M atm^−1^ at 25 °C of Hg^0^)^11^ rapid deposition velocity (5–10 time greater than p-Hg)^12^ and extremely high bioavailability for bacterial methylation. Studies on the workers exposed to low-levels of InHg in a fluorescent light bulb factory suggested that these Hg species may cause a range of dysfunctions such as defects on the monocyte macrophage system^13^, disturbances in the peripheral nervous system and behavioral changes^14^. On the contrary, long term bioaccumulation of low dose Hg^0^ in chloralkali workers is not generally observed due to its rapid excretion largely through urine and stool but also other body fluids at lesser although significant extent^15,16^. Therefore, reported concentrations of Hg above 50 mg kg^−1^ in the hair of chloralkali workers after 40 days of non-exposure time, suggests that the workers are exposed to Hg species which are not easily excreted or washed off the contaminated body parts^17^.

The present study was designed to address the formation of RGM within the facilities of a chloralkali plant. We hypothesise that the observed high Hg concentrations in the hair of chloralkali workers are direct evidence of RGM adsorption on the hair surface caused by its rapid deposition within the plant environment rather than accumulation of Hg^0^ or dietary Hg species.

## Results

### Total Hg in hair and nails

**Figure 1:**
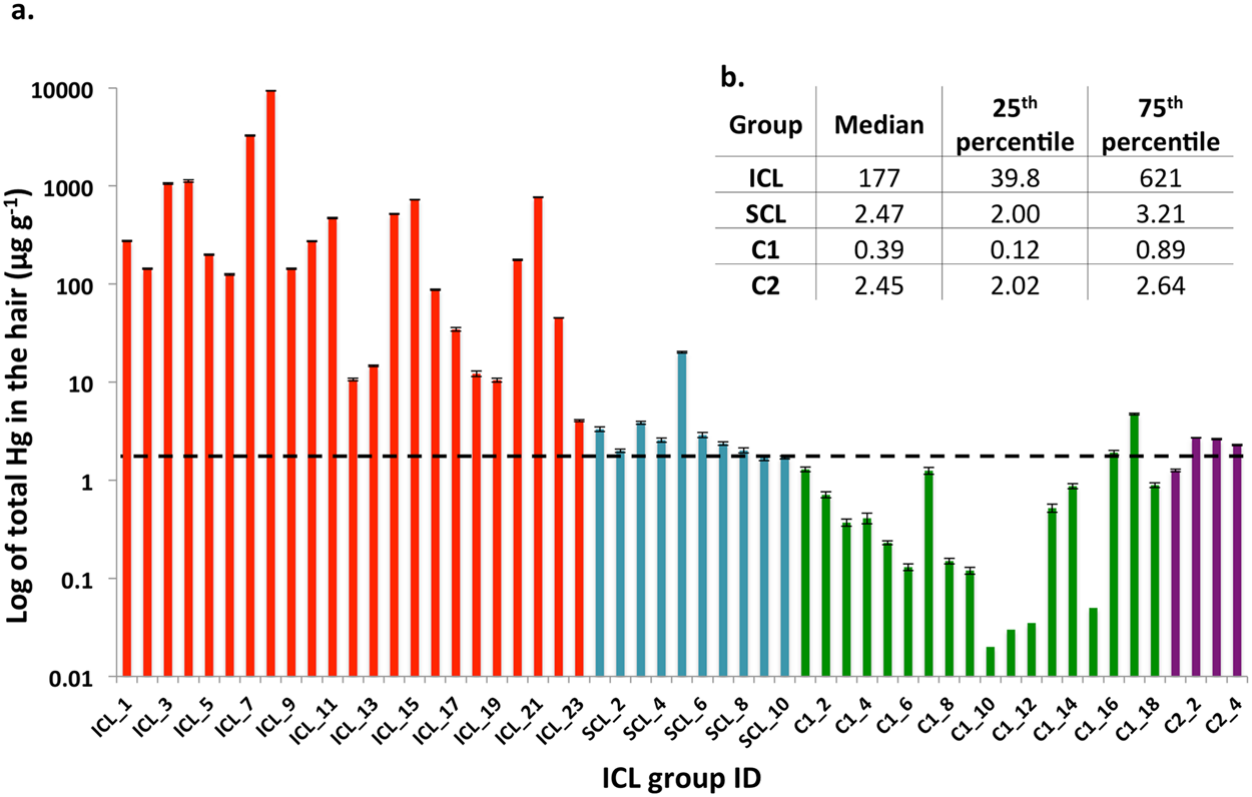
(**a**) Concentration of total Hg (µg g^−1^) in human hair samples (n = 55). The dashed black line indicates the upper limit of the normal Hg concentration in hair of 2.0 µg g^−1^ ^19^. The error bars represent standard deviation between 3 analysed sub-samples (**b**) Calculated median, 25^th^ and 75^th^ percentile of determined total Hg in the studied groups. ICL group: workers in Hg cell technology chloralkali plant, SCL group: workers from chloralkali plant, which ceased Hg cell technology, C1: students and employees at Punjab University in Lahore, C2: people living in the vicinity of the ICL chloralkali plant.

The hair samples from the ICL group (workers in the Hg cell technology chloralkali plant) showed extremely high concentrations of total Hg (n = 23, median 177 µg g^−1^ from a range between 4.06 ± 0.10 µg g^−1^ and 9,341 ± 76 µg g^−1^, Fig. 1, Supplementary Table 1 and 2). The highest concentration (9,341 ± 76 µg of Hg g^−1^) was recorded in the hair sample of a 54 years old worker who had worked in the Hg cell maintenance section for 30 years. The hair concentration for this worker is up to 10 times higher than previously reported values in hair samples^18^ and to our best knowledge this is the highest Hg concentration recorded in human hair to date. This worker consumes more than 250 g week^−1^ of freshwater fish and about 300–900 g week^−1^ of white and brown rice, has no dental fillings and is presently not known to suffer from a medical condition. In addition, the measured concentration of total Hg exceeded 500 µg g^−1^ in 35% of the sampled population, suggesting that these workers are in direct contact with Hg cells and there might be Hg leakage within the plant. The lowest concentration of total Hg (4.06 ± 0.10 µg g^−1^) was recorded in the hair sample of a 25 years old individual who has worked in the ICL plant as an assistant manager for 1.5 years. This worker consumes about 250 g week^−1^ of freshwater fish and about 450 g week^−1^ of white and brown rice and has one amalgam filling. The low Hg concentration recorded in his hair could be a consequence of limited direct contact with Hg cells due his managerial position. The normal value of Hg in the hair is between 1–2 µg g^−1^ for people who consume fish less than once a day and the US EPA estimated a reference dose of 1 µg g^−1^ based on the assumption that MeHg contributes at least 80% of total Hg^19^. It should be noted that the total Hg concentration in the hair of all ICL workers exceeds this value by up to three orders of magnitude in some cases.

Total Hg concentrations in the hair samples of the SCL group (workers from chloralkali plant, which ceased Hg cell technology), are lower in comparison with the ICL group (n = 10, median 2.47 µg g^−1^ of a range between 1.69 µg g^−1^–20.2 µg g^−1^). Although the median value exceeds the upper limit of normal Hg concentration in hair, this small dataset is biased by one single high value as most of the recorded Hg concentrations are only slightly above or below 2.0 µg g^−1^. Significantly lower Hg concentration (p < 0.05) in the hair of the SCL group in comparison with ICL group might be a result of reduced occupational Hg exposure in the SCL plant, which phased out the use of Hg cell technology 8 years ago.

The recorded total Hg concentration in the C1 group (students and employees at Punjab University in Lahore) ranged between below the detection limit (<0.03 µg g^−1^) and 4.73 µg g^−1^ (n = 18). The median of 0.39 µg g^−1^ is significantly lower (p > 0.05) than Hg concentration found in the hair of both ICL and SCL groups. In addition, most of the hair samples in this group contain lower Hg concentrations than the upper limit of normal Hg concentration in the hair.

Similarly, total Hg concentration in the C2 group (people living in the vicinity of the ICL chloralkali plant) ranged between 1.26 µg g^−1^ and 2.71 µg g^−1^ (n = 4). The median concentration of 2.45 µg g^−1^ was significantly lower (p > 0.05) in comparison with the ICL group. However, most of the hair samples in the C2 group showed higher concentrations of total Hg than the upper limit of normal Hg concentration in the hair.

**Figure 2:**
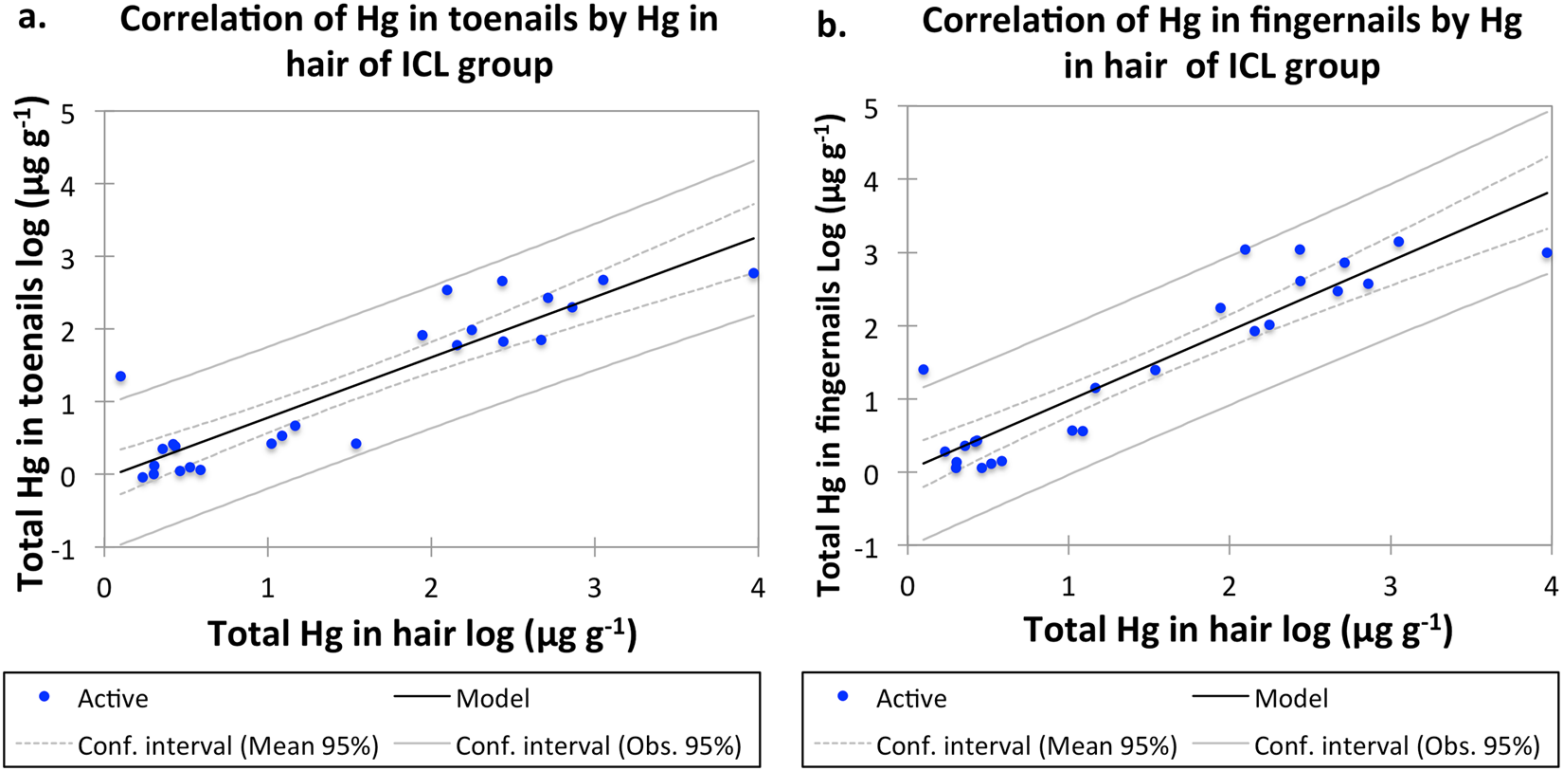
Correlation of total Hg concentration between hair and toenails (**a**) R^2^ = 0.809 m = 0.830 and hair vs. fingernail samples (**b**) R^2^ = 0.838 m = 0.955 belonging to the ICL group. The model correlation line is based on linear regression. m = slope of the linear regression line.

Total Hg concentrations in toenail and fingernail samples collected from the ICL group were extremely high, with the highest values of 590 µg g^−1^ and 1,099 µg g^−1^, respectively (Supplementary Table 1). Previous studies showed higher Hg concentrations in the fingernails in comparison with toenails generally due to direct contact of fingernails with the source of Hg exposure. Additionally, strong positive correlation was observed between total Hg in the hair and toe- and fingernails of the ICL group (Fig. 2a,b, respectively).

### Investigation into the external adsorption of Hg^0^ and HgCl_2_ in human hair

**Figure 3:**
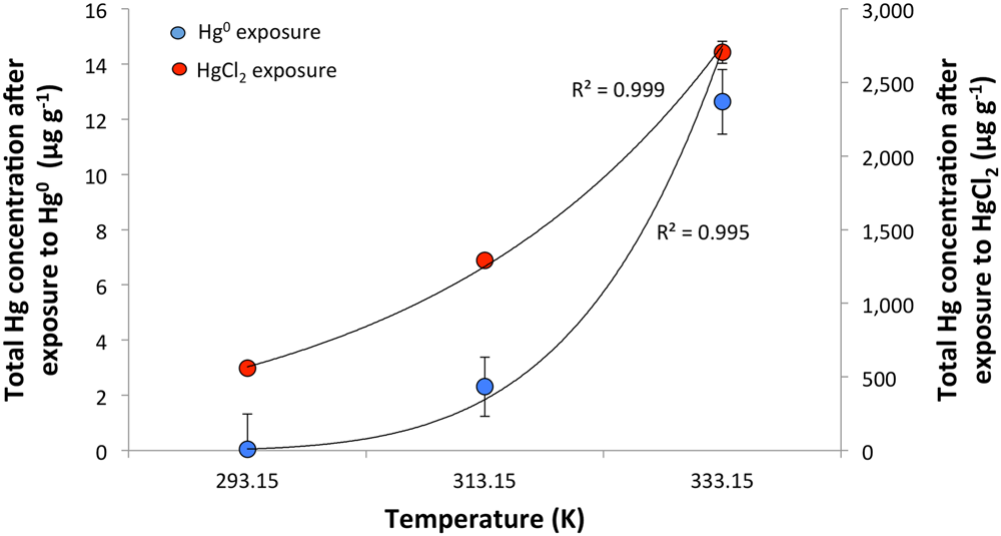
Comparison of total Hg in the hair exposed to Hg^0^ (blue circles) and HgCl_2_ (red circles) under controlled conditions, temperature and time. The error bars represent standard deviation of triplicate measurement of two sub-samples.

Hair exposure simulation to Hg^0^ and HgCl_2_ in closed chambers at different temperatures demonstrated that both Hg species are being taken up by the exposed hair, however at significantly different rates (Fig. 3, Supplementary Table 3).

**Figure 4:**
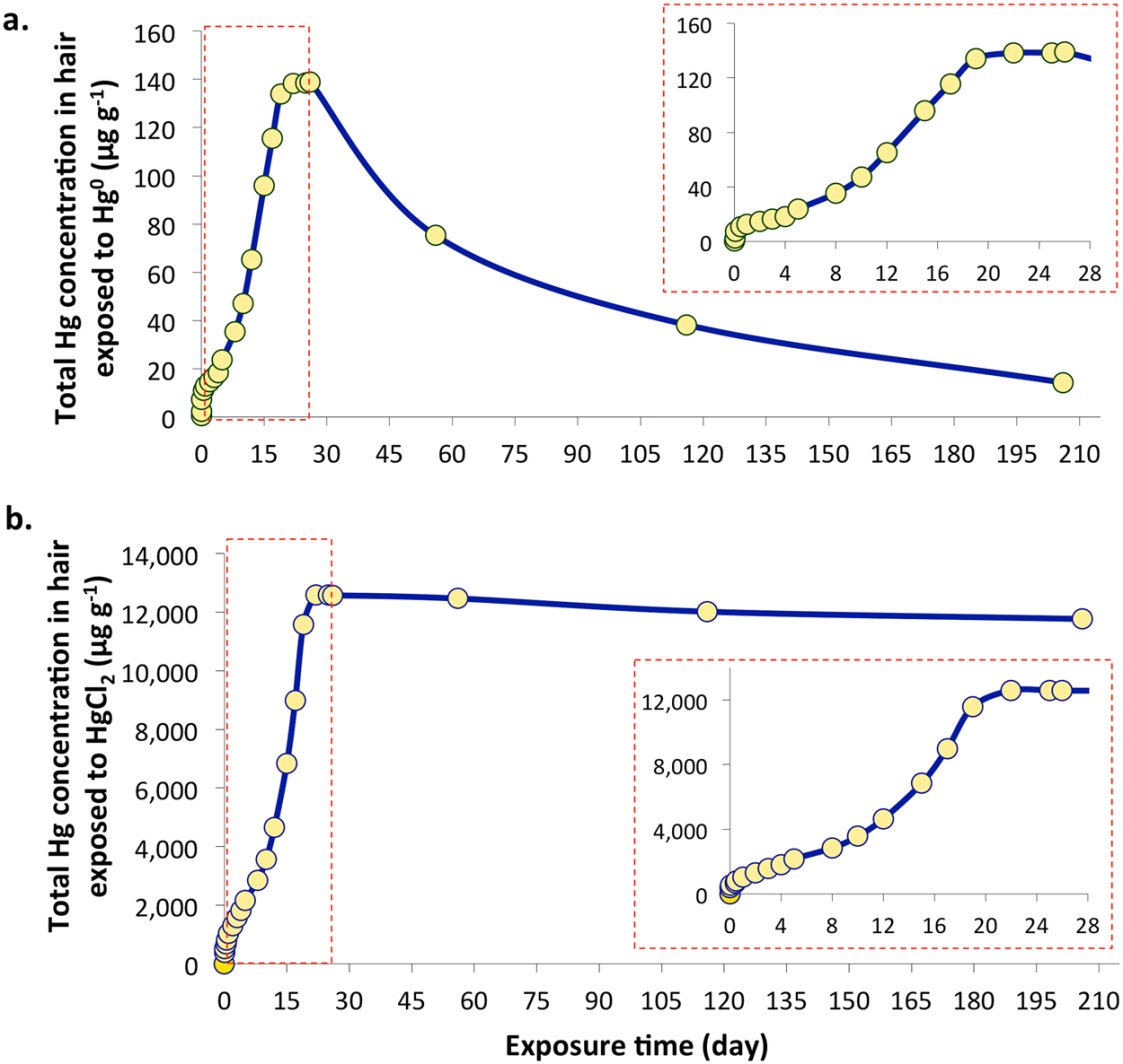
Time related Hg accumulation in the hair exposed to Hg^0^ (**a**) and HgCl_2_ (**b**) at 40 °C (313.15 K°) under controlled conditions for 26 days and subsequent Hg desorption during the relaxation period. The errors expressed as one standard deviation (n = 3 are smaller than the icon for the measurement. n = 3 represents 1 sub-sample collected from 3 parallel exposure experiments.

While the accumulation of Hg^0^ at 20 °C (293.15 K) is very low, interestingly, total Hg concentration in the hair exposed to HgCl_2_ is 4 orders of magnitude higher. The rate of Hg^0^ accumulation steeply increases upon elevating the temperature in the exposure chambers, however the total Hg concentration deposited on the hair exposed to HgCl_2_ in the saturated chamber at 60 °C (333.15 K) is still 2 orders of magnitude higher in comparison with Hg^0^. Laboratory measurements of Hg vapor pressure showed that vapor pressure of Hg^0^ is significantly higher at 23 °C (0.17 Pa)^20^ than the vapor pressure of HgCl_2_ (0.033 Pa)^21^, however with increasing temperature this trend is reversed and at 60 °C the vapor pressure of HgCl_2_ is by 1.29 Pa higher than for Hg^0^ (Table 1). Concomitantly, calculated saturated concentration of Hg in the exposure chambers follows this trend. Therefore, significantly higher Hg concentrations measured in the hair exposed to HgCl_2_ at lower temperature cannot be explained by varying vapor pressure, but rather by a shift in the equilibrium of Hg in the headspace as a consequence of different rates of Hg^0^ and HgCl_2_ adsorption and desorption from the hair surface. HgCl_2_ in the headspace of the exposure chamber is constantly being removed through adsorption on the hair surface and because desorption doesn’t occur or at least at a very slow rate (Fig. 4b), the equilibrium in the headspace is distorted. In order to reach an equilibrium between the solid and gas phase, more Hg enters the gas phase than it is experimentally observed for HgCl_2_ under closed system conditions. As a result of continuous enrichment of the headspace with HgCl_2_, the hair in the exposure chamber accumulated significantly larger concentration of Hg than predicted by the low vapor pressure of HgCl_2_. On the contrary, Hg^0^ desorbs from the hair surface at a much faster rate (Fig. 4a) what not only allows for equilibrium to be reached, but also accounts for the significantly lower concentration of Hg accumulated on the hair surface.

**Table 1:** Vapor pressure and calculated Hg concentrations in the exposure chambers under specified experimental conditions.

Temperature (K)	Hg^0^		HgCl_2_	
	Vapor pressure^a^ (Pa)	Concentration (ng/mL)	Vapor pressure^b^ (Pa)	Concentration^c^ (ng/mL)
296	0.221	18.0	0.033	2.7
313	0.855	65.9	0.800	61.7
332	3.28	238	4.80	349

^a^Data obtained from reference^20^.

^a^Data calculated based on the experimental measurement from reference^21^.

^c^Concentration is calculated for Hg(II).

### Simulation of Hg exposure in the chloralkali plant

The total Hg concentration in hair samples exposed to Hg^0^ at 40 °C for 26 days increased gradually over time from 0.37 ± 0.01 µg g^−1^ to 133.92 ± 0.21 µg g^−1^ during 19 days of exposure (Fig. 4a, Supplementary Table 4). After 19 days, the concentration stabilized for approximately one week (138.24 ± 1.59 µg g^−1^ to 138.89 µg g^−1^). Thereafter, the Hg concentration in the remaining hair samples declined from 138.89 ± 1.59 µg g^−1^ to 14.27 ± 0.57 µg g^−1^ after being left in an open clean vial for the period of six months. This indicates relatively low ability of adsorption of Hg^0^ on the hair most probably due to labile binding of this Hg species which is advocated by fast desorption rate once the hair is removed from the saturated conditions.

Similarly, total Hg in hair samples exposed to HgCl_2_ for a period of 26 days showed a steep increase during the first 17 days of exposure (Fig. 4b, Supplementary Table 4). Subsequently, total Hg in hair exposed to HgCl_2_ stabilized after 20 days for about ten days (12,582 ± 23 µg g^−1^ to 12,576 ± 61 µg g^−1^), however, the concentration of Hg did not changed significantly after the six months relaxation period (12,181 ± 24 µg g^−1^). More interestingly, much higher Hg concentration were found in the hair during the exposure to HgCl_2_ although its vapour pressure at studied temperature (313 K) is similar to the vapour pressure of Hg^0^ (0.800 and 0.855 Pa, respectively). This observation is most probably a result of distorted equilibrium in the HgCl_2_ exposure chamber, where irreversible adsorption of HgCl_2_ on the hair surface forces more HgCl_2_ to enter the vapor phase.

### Spatial distribution of Hg in hair samples

**Figure 5:**
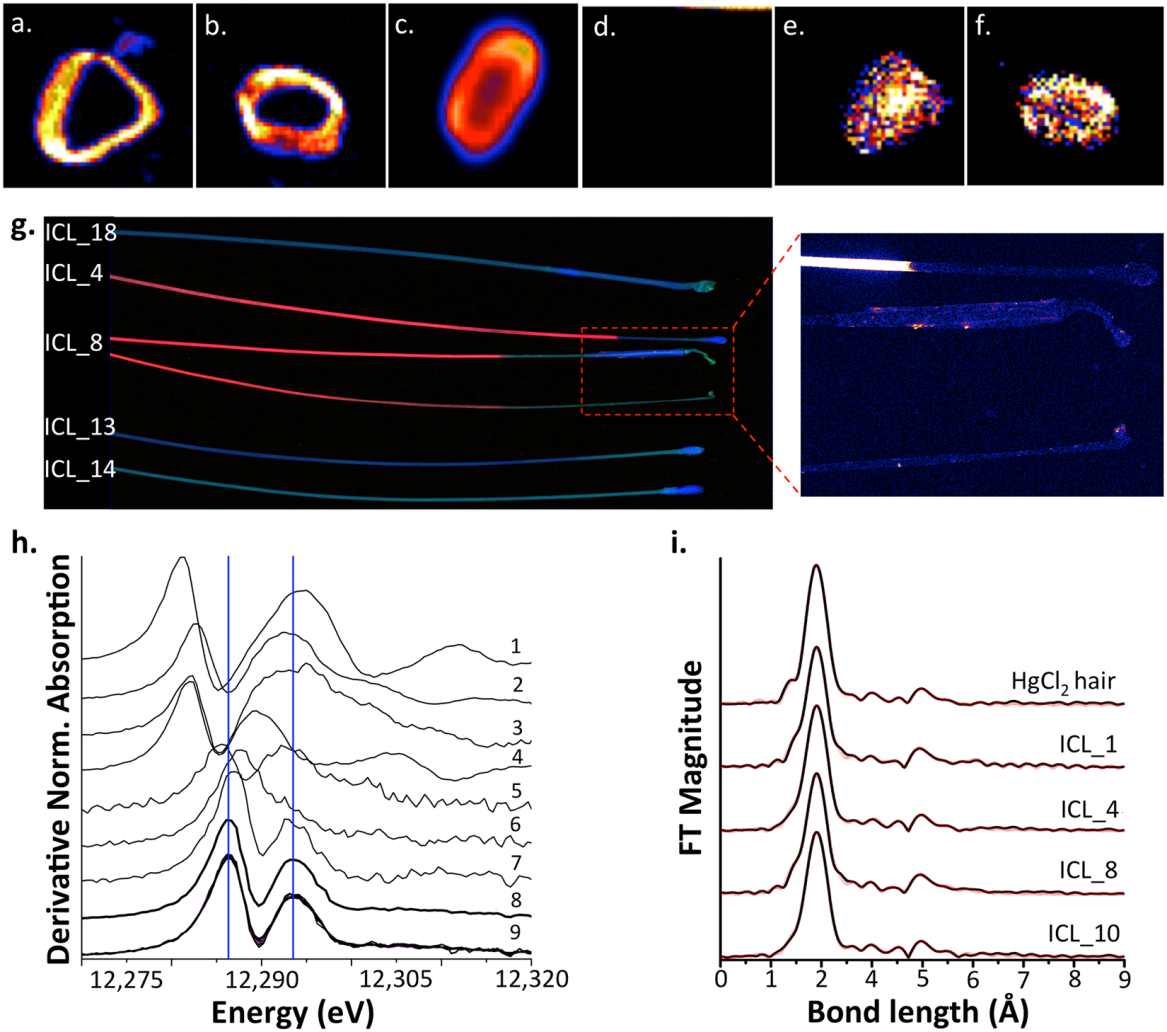
Synchrotron XRF images of hair cross-sections illustrating spatial distribution of Hg (**a**–**d**) and Zn (**e**,**f**) on the hair exposed to HgCl_2_ (**a**,**e**), hair belonging to ICL worker ID 14 (**b**,**f**), hair belonging to ICL worker ID 8 (**c**) and hair exposed to Hg^0^ (**d**). (**g**) XRF generated tomography of Hg (red) and Zn (blue) distribution in the selected hair of ICL workers suggesting adsorption of Hg on the outer layer of the hair. Zoomed in section of the hair from ICL 4 and 8 shows some deposits of Hg in the root of the hair. (**h**) Synchrotron generated XANES L_III_ spectra of Hg in the selected standards and hair/toenail samples (1: Hg^0^, 2: Hg(I) sulfate, 3: Hg(II) sulfate, 4: Hg(II) sulfide, 5: HgCl_2_, 6: MeHgCl, 7: MeHgGS, 8: Hg(GS)_2_, 9: hair and toenails) and calculated bond length between Hg and its bonding atom in the hair exposed to HgCl_2_ and selected hair sample of ICL workers (**i**).

Results from synchrotron generated XRF mapping showed high similarities in Hg distribution between the hair samples obtained from the ICL group and the hair sample exposed to HgCl_2_ (Fig. 5).

It is apparent from the hair cross section that Hg is adsorbed mainly in the cuticle of the hair when exposed to HgCl_2_ (Fig. 5a). Similar pattern in Hg distribution can be seen on the map of ICL worker hair containing 517 ± 4.0 µg g^−1^ (ID ICL_14, Fig. 5b) however, the hair of the ICL worker, which contains extremely high Hg concentrations (9,341 ± 76 µg g^−1^, ID ICL_8, Fig. 5c) produced a slightly different image. This could be explained by oversaturation of the outer hair cuticle and subsequent slow diffusion of Hg to the cortex and medulla of the hair. Distribution of Zn in the hair sample exposed to HgCl_2_ and from ICL worker ID ICL_4 resulted in a very different map as Zn is being homogeneously distributed in the cortex of the hair, most probably in the form of dietary metabolites (Fig. 5e and f). This observation verifies that the high Hg concentrations present in the hair are of an atmospheric rather than dietary origin. On the contrary, Hg levels in the hair exposed to Hg^0^ (Fig. 5d) were below the instrumental LOD most probably as a result of Hg desorption during the relaxation period of the exposure experiment.

It appears that the majority of examined hair by XRF mapping were in the anagen phase however, the disfigured roots of ICL 8 suggests that these hair were in the talogen stage (Fig. 5g). XRF images also confirmed that a large majority of the Hg (red colour on Fig. 5g) is localized in the part of the hair (ICL_4 and ICL_8) that has emerged from the scalp i.e. most of the Hg is due to external adsorption. Moreover, the magnified image of hair root samples depicted Hg on the outer root sheath and in the bulb. This indicates that some of the Hg is possibly coming in through blood circulation and is excreted by the sebaceous gland. However, some Hg deposits were also found on the outer edge of the hair root, which is below the point of hair emergence from the scalp.

### Hg speciation in hair and toenail samples

Linear combination fitting of Hg L_III_- spectra showed that most of the Hg exists as Hg(II) and considering the similarity of the XANES spectra, the local structures around the Hg atoms seem to be similar to those in Hg bound to glutathione (Hg(GS)_2_) in both the hair and toenails of the ICL group (Fig. 5h). As the absorption bands for the HgCl_2_ standard are shifted to lower energies, it is highly improbable that Hg species on the hair are in the form of HgCl_2_. However, the Fourier transformed k^3^-weighted L_III_-edge EXAFS spectra of 4 hair samples showed very similar distance between Hg and its nearest scattering atom, most probably a sulfur atom, suggesting that ICL workers were exposed to the identical Hg species (Fig. 5i). Moreover, the comparison of the bond length of Hg to the nearest ligand between the hair belonging to the ICL group with HgCl_2_ exposed hair showed a very high degree of similarities.

**Figure 6:**
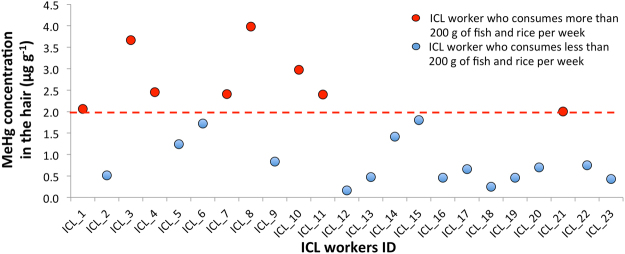
Comparison of MeHg in hair (µg g^−1^) per individual ICL worker with respect to their fish and rice consumption. The red dashed line indicates the upper limit of the normal Hg concentration in the hair (2 µg g^−1^). The error bars express one standard deviation between 2 analysed sub-samples are smaller than the icon for the measurement. The ICL group represents the workers in the Hg cell technology chloralkali plant.

The median concentration of MeHg in the hair samples of ICL workers determined by GC-ICP-MS was 1.24 µg g^−1^, varying from 0.17 to 3.98 µg g^−1^ (Supplementary Table 1), which is significantly lower (p < 0.05,) than the median of the InHg concentration of 176 µg g^−1^ ranging from 3.67 to 9336 µg g^−1^. Although MeHg accounted for a relatively low fraction of the total Hg (median 0.6%), it has to be mentioned that more than 40% of analysed hair samples contained MeHg above the upper limit of normal Hg concentration (2.0 µg g^−1^). Moreover, according to the questionnaires filled in by the participants at the time of the hair samples collection, it was found that MeHg concentrations elevated above 2.0 µg g^−1^ in the hair, belonged to workers who consumed more than 200 g of fish per week (Fig. 6).

## Discussion

The results obtained from this study suggest that the chlor-alkali workers were exposed to HgCl_2_ or other RGM rather than Hg^0^. Following the exposure, these Hg species are deposited on the outer surface of the hair resulting in extremely high concentrations of Hg found in the hair of ICL workers. The results also suggest, that after the deposition of HgCl_2_ on the hair surface, Hg binds extremely strongly to bio-thiols present in the hair and its desorption doesn’t take place even after prolong period without exposure. These main findings are discussed and argued below.

Hair biomarkers are generally used for monitoring bioaccumulation of dietary MeHg and previous studies showed that in general about 90% of internally deposited Hg in the hair is in its methylated form^22^. Human exposure to the lethal dose of MeHg resulted into Hg hair concentration of 1,100 µg g^−1^ after 21.8 days following the incident^23^ therefore considering the extremely high concentration of total Hg found in the hair of ICL workers, (max of 9341 ± 76 µg of Hg g^−1^) the possibility of internal deposition of dietary MeHg was excluded. Similarly, only a small fraction of inhaled inorganic Hg is being deposited in the hair, whereas the larger portion remains in the blood stream and undergoes re-distribution through the body^24,25^. Therefore, we hypothesise that Hg present in the hair of ICL workers is adsorbed on the outer surface of the hair. Interestingly, we also observed a significant correlation between total Hg in the hair and the finger- and toenails, R^2^ = 0.809 and 0.838, respectively. In general, external adsorption shows significant correlation of Hg in the body parts, which are fully exposed to the outer environment. The workers responsible for the cell’s maintenance and clean up are required to enter the redundant cells and manually clean them from the process residues, often wearing sandals without any protection. During this process, the toenails are fully exposed to Hg residues leading to strong binding of Hg on the surface of the nails.

Interestingly, the Hg concentrations in the hair of SCL workers who are not using Hg cell technology for the past 8 years were elevated, however, significantly lower than in ICL workers. As these workers are no longer exposed to Hg vapor it is possible that the increased concentrations are remnants of historical adsorption of Hg in the body. Following the period of exposure to high Hg concentrations, it may take more time for the body to slowly remove Hg through the blood stream which will also lead to re-distribution of Hg to the hair. The amount of deposited Hg in the hair may be amplified by the uptake of dietary MeHg, which is also deposited in the hair. Another possible explanation may be an exposure of the workers to the historical deposits of HgCl_2_ within the plant, which although ceased using Hg-cells but did not achieve full decontamination of the environment from Hg. Additionally, our results showed an increased risk of Hg exposure of the population living in the vicinity of the ICL chloralkali plant (control group 2) as their total hair Hg concentration was significantly higher in comparison with the control group 1. Although the Hg species emitted from the ICL plant are not known, it is most likely that the immediate environment is exposed to RGM rather than Hg^0^ due to its high dry deposition velocity. According to Landis *et al*., the RGM/Hg^0^ ratio decreased from 2.1% to 1.3% within the distance of 800 m from the vents of the chloralkali plant, resulting from rapid depositing of RGM^9^.

Our results of the simulated hair exposure study indicate that HgCl_2_ adsorbs on the hair surface at a significantly higher rate than Hg^0^ at the studied temperatures. This observation suggests firstly that HgCl_2_ is most likely in the form of RGM, evidenced by an adsorption potential that could explain the extreme levels of Hg in the hair of the chloralkali plant workers. Secondly, it is very reactive and binds strongly to reduced sulphur in the hair, whereas the hair exposed to Hg^0^ releases most of Hg again after the exposure. It cannot be excluded that if present, some Hg^0^ could be removed from the hair surface during the washing step. But, while several studies concerning Hg removal from the hair during the washing procedures were published^26,27^, these studies considered only the exposure to HgCl_2_ and data for Hg^0^ are not available. Nonetheless, the results from our long-term exposure experiment showed that Hg^0^ undergoes slow desorption after the source of emission is removed. Therefore, considering that the collected hair samples were analysed 30 days after the collection, the Hg concentration found on the hair should be much lower if the workers were exposed to Hg^0^. Similarly, Hg levels found in the hair of SCL workers also corroborate exposure to RGM. Moreover, the actual amount of Hg adsorbed on the hair exposed to the fully saturated Hg^0^ vapor, is up to two orders of magnitude lower than the amount of Hg found in the hair of some ICL workers. Thus, it is very improbable that solely Hg^0^ is present in the air of the chloralkali plant.

XRF mapping showed clear accumulation of Hg on the outer surface of the hair belonging to the ICL workers, which agreed with the distribution pattern from the hair exposed to HgCl_2_ thus further advocates that ICL workers were exposed to RGM. Additionally, the XRF generated tomography also indicated that Hg is accumulated in the section of the hair after it emerged from the scalp and therefore it was deposited externally. The comparison of the bond length between Hg and its neighbouring atom (Fig. 5i) showed a very high degree of similarity between the hairs belonging to ICL group and HgCl_2_ exposed hair. This observation suggests that the hair of ICL workers were exposed HgCl_2_ or similar RGM. However, because the absorption bands for the HgCl_2_ standard were shifted to lower energies (Fig. 5h), the compounds that are actually present on the surface of the hair are not in the form of HgCl_2_. Significant similarity in the absorption bands can be observed between the hair and nails of ICL workers and Hg(GS)_2_ standard providing additional evidence that formation of Hg containing biothiols takes place.

To identify the impact of high Hg occupational exposure on the MeHg accumulation, we conducted Hg speciation on the ICL workers’ hair. The data revealed that MeHg concentration in the hair of 8 workers was above the safety limit of the WHO, however, according to the questionnaires filled in by the participants, it was found that these workers consumed more than 200 g of fish or rice per week. Thus it can be concluded that Hg deposited within the inner part of the hair has predominantly dietary origin such as fish consumption, while the deposition of Hg on the outer surface of hair originated from the atmospheric pollution of Hg cell operating chloralkali plant.

## Conclusion

While the previously reported studies assumed that chloralkali workers are predominantly exposed to the Hg^0^ vapor, our study clearly demonstrated the formation of reactive gaseous Hg and its external adsorption on the exposed body parts. We have reported the highest hair Hg concentration found to date 9,341 ± 76 µg g^−1^, which is a direct result of continuous occupational exposure. Our suggestions of RGM formation are supported by strong evidence obtained from the simulated exposure experiments, which indicate extremely high absorption potential of HgCl_2_, its very fast rate of adsorption and formation of irreversible bond on the surface of the exposed hairs and nails. Exposure to such a highly reactive Hg species may lead to unexpected adverse health effects not only to the workers but also the immediate environment as a result of continuous scavenging of essential biothiols by RGM.

## Materials and Methods

### Sample collection

The studied population consisted of 56 participants from the Punjab province who had volunteered for an exposure examination and in total 111 biological (hair & nails) samples were analyzed. The sampled population was divided into four groups as follows: ICL group (workers from the Hg cell chloralkali plant) provided 23 hair, 18 toenail and 16 fingernail samples (n = 57). SCL group (workers from chloralkali plant which phased out Hg cell technology 8 years before sampling) provided 10 hair, 6 toenail and 6 fingernail samples (n = 22). C1 group (control group; students and staff in Punjab university, Lahore, Pakistan) provided hair samples (n = 18). C2 group (control group 2; people living in the vicinity of the ICL chloralkali plant) provided 4 hair, five 5 toenail and 5 fingernail samples (n = 14)

For a general lifestyle and health examination a questionnaire had been provided by TESLA (Trace Element Speciation Laboratory Analysis, University of Aberdeen), which contained general characteristics of the subjects such as duration of the employment in the plant, amount and frequency of fish and rice consumption, known health conditions, number of amalgam filling. Complete questionnaires can be found in the Supplementary Information S5.

All experiments described in this manuscript were approved by the Ministry of Environment, Government of Pakistan and the methods were carried out in accordance with the guidelines and regulations provided by the Ministry of Environment, Government of Pakistan. An informed consent was obtained for study participation and publication of identifying information/images from all human subjects participating in the study.

### Sample treatment

In order to eliminate any surface contaminants and to normalize the background values of the individual biological samples, they were sonicated for 1 h in 100 mL of a detergent solution (1% RBS 25 detergent in deionised water) and then rinsed 4 times with 100 ml of deionised water. Rinsed samples were dried at 50 °C overnight and left to equilibrate with atmospheric humidity for 5 hours prior to storage^28^.

### Simulated hair exposure study

An exposure study was designed to simulate the environmental conditions in the chloralkali plant, which could help to identify Hg species dominantly present in the air of the plant. For this purpose hair samples were collected from a 44 years old male (volunteer) and washed following the previously defined washing procedure. Hg concentration in the hair was determined (1.02 ± 0.01 μg g^−1^) prior to the exposure experiment to allow for baseline correction of the exposed hair. For the exposure experiment an aliquot of accurately weighted (∼0.3 g) hair samples collected from the volunteer were placed in triplicates into 500 mL atmospheric adsorption chambers, each containing about two drops of Hg^0^ or two crystals of HgCl_2_. Adsorption chambers were kept at constant temperature of 20 °C (293.15 K) in the water bath for 8 hours per day for one week. During the night, the chambers were left at room temperature in a fume cupboard. Afterwards the bath temperature was increased to 40 °C (313.15 K) and the heating cycle was repeated. During the last heating cycle, the water bath temperature was kept at 60 °C (333.15 K). At the end of each cycle, one sub-sample (∼0.015 g) of exposed hair was collected, washed and analysed as describe above.

For the long-term exposure study, accurately weighted (∼3.5 g) hair samples from the same volunteer were kept in the adsorption chambers at a constant temperature of 40 °C (313.15 K) for 26 days maintaining the same heating cycle (8 h day^−1^). Afterwards, the remaining hair samples were left exposed to ambient atmosphere (relaxation period) for six months. The sampling points were as follows: 30 min, 1 h, 3 h, 5 h and 24 h on day 1; once every other day for days 2–5; every 2–3 days for the remaining 21 days. Thereafter, the sampling was done after 1, 3 and 6 months of the relaxation period. Collected samples were washed and analysed as describe above.

### Total Hg analysis

To address the total Hg concentration in the collected samples and experimentally exposed hair, an aliquot of accurately weighted (∼10 mg) hair or nail sample was pre-digested in 5 ml of HNO_3_ for 20 minutes. Autoclave digestion was performed in closed glass vessels for 90 minutes at 100 °C in a water bath. Digests were stored at 4 °C until further use.

Cold vapour atomic fluorescence spectroscopy (PS Analytical Ltd, UK) was used for total Hg analysis. The digested samples were diluted to 5% acid concentration prior to measurement. The diluted digest was first mixed with the blank solution (5% HNO_3_), followed by reaction with reductant (2% SnCl_2_) in the sample valve. This reaction resulted in vapour generation of elemental Hg, which was purged with argon gas from the gas-liquid separator through the dryer into the atomizer.

The instrument was calibrated using aqueous Hg standards, and a calibration was performed on each measurement day by using standard solutions in the range of 0.02 to 5 ng g^−1^. Certified reference materials (CRM), NIES-13 and IAEA-085 (human hair) were used during each digestion cycle to validate the analytical method, which was also verified by spiking of the method blank and the reference materials with known concentration of HgCl_2_ and MeHgCl. All CRM samples were treated the same way as investigated hair samples. The measured concentration in the CRMs was in satisfactory agreement with certified values and percentage recoveries were 99.8% for NIES-13 and 99.9% for IAEA-085 (n = 6). The percentage recovery of spiked HgCl_2_ ranged between 94.9 and 102.8% (n = 12) and the recovery of MeHgCl was in the range of 97.1–98.6% (n = 12). Hg concentration in the methods blank was below the instrumental limit of detection (LOD), 0.01 ng g^−1^, which was defined as 3 times the standard deviation of 10 measurements of the calibration blank.

### Statistical methods

XLSTATPro was used for the statistical comparison of total Hg concentrations between sampled groups and 2 way ANOVA tests were executed and relationships with P values < 0.05 were quoted as significant.

### MeHg quantification

To determine the fraction of total Hg in the collected samples, which originated from the dietary exposure to MeHg, the hair and toenail samples were subjected to Hg speciation analysis. For this purpose an aliquot of accurately weighted (∼50 mg) hair or nail sample was spiked with enriched Me^201^Hg and led to equilibrate for one hour. Afterwards the samples were manually shaken for 1 min and 3 mL of HCl (4%, m/m) was added into each sample, which were mechanically shaken for 2 min. Samples were centrifuged at 3749 × g for 20 min and the supernatant was collected. The leaching process was repeated with 2 mL of HCl (4%, m/m) and the combined supernatants were made up to 5 mL with ultrapure water. One mL from the leachate was buffered with 5.0 mL of acetate buffer (0.1 M, pH 3.9) and the pH was adjusted to 3.9. One mL of iso-octane was added to the mixture followed by 1 mL of 1% NaBPr_4_. Samples were manually shaken for 5 min and centrifuged at 3749 × g for 10 min. The organic layers were transferred into amber GC vials and stored at −20 °C until analysis by GC-ICP-MS.

Analysis was performed using a HP-6890 gas chromatograph (Agilent Technologies, USA) hyphenated to 7500c ICP-MS (Agilent Technologies, USA) via a heated silcosteel^®^ transfer line (Thames Restec, UK). The intensities of *m/z* ^199^Hg, ^200^Hg, ^201^Hg, ^202^Hg, ^203^Tl, and ^205^Tl were monitored in the transient signal mode and all integrated peak areas were mass bias corrected.

ICP-MS was manually tuned on each day of analysis using homemade tune solution. The analytical performance of the developed method was tested on NIES-13 CRM, which is certified for MeHg concentration. Additionally, NIES-13 was also spiked with known MeHgCl concentration and both tests resulted in satisfactory mean recoveries of 94.2 and 97.6% respectively (n = 6). The LOD, defined as S/N = 3 was 0.12 ng g^−1^ and all analysed method blank contained Hg below the calculated LOD.

### Hg speciation in the solid hair and nail samples

#### X-ray absorption near edge spectroscopy (XANES) analysis

XANES analysis provides unique information about the electronic structure of measured the element and thereby can provide a unique fingerprint of the type of chemical species the element is found in. This analysis was used to identify the Hg species present on the surface of the sampled and experimentally exposed samples.

XANES measurements were conducted at the X-ray absorption spectroscopy (XAS) beamline at the Australian Synchrotron (Melbourne, Australia). The storage ring operated at 3 GeV in top-up mode. A liquid N2 cooled double crystal Si (111) monochromator was used to select the incident photon energies and a platinum-coated mirror was used for harmonic rejection. XANES spectra were collected in fluorescence mode using a 100-element solid state Ge detector. The beam size was approximately 250 × 1500 μm and the samples were analysed as ground material. Calibration was performed by assigning the first derivative inflection point of LIII edge of gold foil (11919 eV) with periodic recalibration to ensure stability.

The collected spectra were analyzed using the Athena software program in the computer package IFEFFIT^29^ for data reduction and WinXAS 3.0^30^ for data fitting. The individual spectra for each sample were averaged followed by subtraction of the background through the pre-edge region using the Autobk algorithm^31^ and normalized to the atomic absorption of one. The data were converted from energy to photoelectron momentum (*k*-space) and weighted by *k*^3^ using WinXAS. Extended X-ray absorption fine structure spectra were calculated over a typical *k*-space range with a Bessel window. Fourier transforms were performed to obtain the radial distribution function in *R*-space. Plotted *R*-space (Å) data are not phase shift corrected so that the true distances are between 0.3 and 0.5 Å longer than the distances shown. Structural parameters were extracted with fits to the standard XAFS equation. Using the FEFF8 and ATOMS codes^32,33^, *ab initio* amplitude and phase functions for single shells were calculated for HgCl_2_^34^ and Hg(cysteaminate)_2_ crystal structures^35,36^ following procedures outlined by Mah and Jalilehvand^37,38^. Structural parameters for Hg interaction were obtained by least-squares refinements of the theoretical model function χ (k), allowing R, σ^2^, and ∆E_0_ to float, to the *k*^3^-weighted EXAFS oscillation over the *k*-range of 2.1–13 Å^−1^, after Fourier-filtering in the range of 1.20–3.40 Å. The S_0_^2^ value was held constant at 1.0 to allow refinements of the coordination number. The accuracy of the mean bond distance R is estimated to be within ±0.02 Å and the coordination number N is estimated to be accurate to ±20%, based on the results of theoretical fits to spectra of reference compounds of known structure.

#### X-ray fluorescence elemental mapping

Samples were placed between two layers of 4 µm thick Ultralene film and analysed at the X-ray Fluorescence Microscopy (XFM) beamline at the Australian Synchrotron^39^. This undulator beamline is equipped with a Si (111) monochromator and Kirkpatrick-Baez mirrors focusing the beam to a spot size of approximately 2 μm^2^. Elemental maps were collected at 15.5 keV using a 384-element Maia detector in a backscatter geometry^40^. The samples were analysed continuously in the horizontal direction (‘on the fly’) with steps of 2 μm in the vertical direction. The transit time was approximately 8 ms per pixel. The full XRF spectra were then analysed using GeoPIXE^41,42^. This software uses Dynamic Analysis to subtract background and resolve overlapping peaks when generating elemental maps, thus allowing calculation of semi-quantitative values for all the different elements.

## Electronic supplementary material

Supplementary information
