# Supplementary material for: Reactive gaseous mercury is generated from chloralkali factories resulting in extreme concentrations of mercury in hair of workers
Source: Sci Rep. 2018 Feb 27;8:3675. doi: 10.1038/s41598-018-20544-5 (PMC5829213; doi:10.1038/s41598-018-20544-5)
Supplement: Supplementary file 1 — Supplementary information [file 41598_2018_20544_MOESM1_ESM.doc]

**Supplementary Material**

**Reactive gaseous mercury is generated from chloralkali factories resulting in extreme concentrations of mercury in hair of workers**

Abdelkarem A. S. Elgazali,^1^ Zuzana Gajdosechova,^1^ Zaigham Abbas,^2^ Enzo Lombi,^3^ Kirk G. Scheckel,^3,4^ Erica Donner,^3^ Heidelore Fiedler^5,6^, Jörg Feldman,^1^ Eva M. Krupp^1*^

^1^Trace Element Speciation Laboratory, University of Aberdeen, Department of Chemistry, Aberdeen, UK, ^2^ Government of Pakistan Ministry of Climate Change, LG & RD Complex, Islamabad, Pakistan, ^3^Future Industries Institute, University of South Australia, Building X, Mawson Lakes Campus, South Australia. ^4^United States Environmental Protection Agency, National Risk Management Research Laboratory, Cincinnati, OH  45224, USA, ^5^United Nations Environmental Programme, Chemicals Branch, DTIE, 11-13 Chemin des Anemones, CH-1219 Chatelaine, Switzerland. ^6^Present address: Örebro University, School of Science and Technology, MTM Research Centre, SE-701 82 Örebro, Sweden.

Address correspondence to Jörg Feldmann or Eva M. Krupp, Trace Element Speciation Laboratory, University of Aberdeen, Department of Chemistry, Meston Walk, Aberdeen, AB24 3UE, UK, Phone: +44 (0)1224 272901, Email: [j.feldmann@abdn.ac.uk](mailto:j.feldmann@abdn.ac.uk) or [e.krupp@abdn.ac.uk](mailto:e.krupp@abdn.ac.uk)

**Supplementary Table 1.** Total Hg and MeHg concentration in µg g^-1^ ± SD (n = 12) in the hair and toe- fingernails of ICL workers.

|  | **Total Hg** | | | **MeHg** |
| --- | --- | --- | --- | --- |
| **Sample ID** | **Toenails** | **Fingernails** | **Hair** | **Hair** |
| ICL_1 | 68.8 ± 0.35 | 405 ± 2.84 | 275 ± 3.6 | 2.07 ± 0.03 |
| ICL_2 | 60.1 ± 0.45 | 83.5 ± 1.78 | 143 ± 1.7 | 0.515 ± 0.01 |
| ICL_3 | NA | NA | 1057± 17.4 | 3.67 ± 0.03 |
| ICL_4 | 472 ± 3.87 | 1402 ± 9.19 | 1124± 32.3 | 2.46 ± 0.03 |
| ICL_5 | 76.3 ± 0.71 | NA | 199 ± 2.46 | 1.24 ± 0.03 |
| ICL_6 | 343 ± 1.97 | 1098 ±12.62 | 125 ± 2.0 | 1.72 ± 0.02 |
| ICL_7 | NA | NA | 3261± 39 | 2.41 ± 0.03 |
| ICL_8 | 590 ± 5.18 | 1000 ± 17.57 | 9341 ± 76 | 3.98 ± 0.03 |
| ICL_9 | NA | NA | 143 ± 2.0 | 0.837 ± 0.01 |
| ICL_10 | 455 ± 8.51 | 1099 ± 42.79 | 272 ± 3.0 | 2.98 ± 0.04 |
| ICL_11 | 71.3 ± 0.53 | 296 ± 11.49 | 470 ± 7.0 | 2.40 ± 0.02 |
| ICL_12 | NA | NA | 10.6 ± 0.3 | 0.166 ± 0.003 |
| ICL_13 | 4.7 ± 0.05 | 14.1 ± 0.33 | 14.7 ± 0.3 | 0.474 ± 0.01 |
| ICL_14 | 270 ± 0.45 | 726 ± 6.34 | 517 ± 4.0 | 1.42 ± 0.02 |
| ICL_15 | 199 ± 1.50 | 371 ± 5.76 | 725 ± 7.0 | 1.80 ± 0.01 |
| ICL_16 | 82.8± 1.92 | 173 ± 6.91 | 87.7 ± 1.6 | 0.461 ± 0.02 |
| ICL_17 | 2.6 ± 0.06 | 24.6 ± 0.12 | 34.5 ± 1.4 | 0.659 ± 0.02 |
| ICL_18 | 3.4 ± 0.07 | 3.6 ± 0.03 | 12.2 ± 0.8 | 0.249 ± 0.01 |
| ICL_19 | 2.7 ± 0.06 | 3.7 ± 0.08 | 10.5 ± 0.5 | 0.461 ± 0.01 |
| ICL_20 | 96.9 ± 1.43 | 103 ± 1.48 | 177 ± 3.0 | 0.702 ± 0.01 |
| ICL_21 | NA | NA | 768 ± 7.0 | 2.01 ± 0.02 |
| ICL_22 | NA | NA | 45.1± 0.3 | 0.751 ± 0.02 |
| ICL_23 | 3.8 ± 0.08 | 4.0 ± 0.10 | 4.06 ± 0.10 | 0.430 ± 0.01 |

NA – samples not available

**Supplementary Table 2.** Total Hg concentration in µg g^-1^ ± SD (n = 12) in the hair of SCL workers and two control groups.

| **Sample ID** | **Total Hg** |  | **Sample ID** | **Total Hg** |
| --- | --- | --- | --- | --- |
| SCL_1 | 3.32 ± 0.18 |  | C1_7 | 1.25 ± 0.10 |
| SCL_2 | 2.00 ± 0.08 |  | C1_8 | 0.15 ± 0.01 |
| SCL_3 | 3.86 ± 0.14 |  | C1_9 | 0.12 ± 0.01 |
| SCL_4 | 2.57 ± 0.13 |  | C1_10 | 0.02 ± NA |
| SCL_5 | 20.2 ± 0.40 |  | C1_11 | 0.030 ± NA |
| SCL_6 | 2.89 ± 0.17 |  | C1_12 | 0.035 ± NA |
| SCL_7 | 2.36 ± 0.09 |  | C1_13 | 0.52 ± 0.05 |
| SCL_8 | 2.01 ± 0.12 |  | C1_14 | 0.87 ± 0.05 |
| SCL_9 | 1.69 ± 0.11 |  | C1_15 | 0.05 ± NA |
| SCL_10 | 1.71 ± 0.06 |  | C1_16 | 1.91 ± 0.10 |
| C1_1 | 1.29 ± 0.07 |  | C1_17 | 4.73 ± 0.11 |
| C1_2 | 0.71 ± 0.05 |  | C1_18 | 0.89 ± 0.05 |
| C1_3 | 0.37 ± 0.03 |  | C2_1 | 1.255 ± 0.04 |
| C1_4 | 0.41 ± 0.05 |  | C2_2 | 2.705 ± 0.01 |
| C1_5 | 0.23 ± 0.01 |  | C2_3 | 2.62 ± 0.04 |
| C1_6 | 0.13 ± 0.01 |  | C2_4 | 2.275 ± 0.04 |

**Supplementary Table 3.** Total Hg concentration (mean ± SD, n = 6) adsorbed on the outer surface of the hair during the controlled exposure experiment. The final Hg concentration in the exposed hair is corrected for Hg found in the hair prior to exposure.

| **Exposure**  **temperature (K)** | | **Total Hg concentration in exposed hair (µg g^-1^)** | |
| --- | --- | --- | --- |
|  |  | **Hg^0^ exposure** | **HgCl_2_ exposure** |
| 293 | | 0.05 ± 0.01 | 559 ± 5.00 |
| 313 | | 2.31 ± 0.01 | 1292 ± 5.00 |
| 333 | | 12.6 ± 0.16 | 2704 ± 74.3 |
| **Total Hg concentration in unexposed hair sample** | | | |
| Control | 0.96 ± 0.03 | | |

**Supplementary Table 4.** Total Hg concentrations in µg g^-1^ (mean ± SD, n = 6) externally adsorbed on the outer surface of the hair during the controlled exposure experiment at 313 K (40 °C).

| **Exposure time** | **Total Hg concentration** | |
| --- | --- | --- |
|  | **Hg^0^ exposure** | **HgCl_2_ exposure** |
| 30 min | 0.37 ± 0.01 | 380 ± 1.65 |
| 1 h | 0.96 ± 0.03 | 526 ± 1.12 |
| 3 h | 2.39 ± 0.01 | 682 ± 0.92 |
| 5 h | 11.0 ± 0.06 | 806 ± 1.80 |
| 24 h | 12.8 ± 0.06 | 1047 ± 11.0 |
| 2 days | 14.7 ± 0.28 | 1297 ± 12.1 |
| 3 days | 16.4 ± 0.26 | 1573 ± 25.1 |
| 4 days | 18.3 ± 0.34 | 1815 ± 13.7 |
| 5 days | 23.7 ± 0.21 | 2167 ± 28.5 |
| 8 days | 35.4 ± 0.17 | 2839 ± 21.1 |
| 10 days | 47.1 ± 0.49 | 3565 ± 16.1 |
| 12 days | 65.3 ± 0.21 | 4648 ± 30.4 |
| 15 days | 95.9 ± 0.39 | 6843 ± 36.6 |
| 17 days | 115.6 ± 0.36 | 8987 ± 125 |
| 19 days | 134 ± 0.21 | 11580 ± 33.9 |
| 22 days | 138 ± 1.59 | 12582 ± 22.6 |
| 25 days | 138± 0.97 | 12586 ± 5.75 |
| 26 days | 139 ± 1.15 | 12577± 61.3 |
| **Total Hg concentration left in exposed hair samples during 6 months relaxation period** | | |
| **Relaxation time** | **Hg^0^ exposure** | **HgCl_2_ exposure** |
| 1 month | 77.3 ± 2.26 | 12516 ± 72.8 |
| 3 months | 38.3 ± 0.81 | 12218 ± 68.6 |
| 6 months | 14.3 ± 0.57 | 12181 ± 24.0 |
| **Total Hg concentration in unexposed hair sample** | | |
| Control | 0.97 ± 0.01 | |

**Supplementary Table 5. Questionnaire filled in by the subjects of the study.**

| **Group No 1: ICL chloralkali workers** | | | | | | | | | | | | | | | | | |
| --- | --- | --- | --- | --- | --- | --- | --- | --- | --- | --- | --- | --- | --- | --- | --- | --- | --- |
| **Sample ID** | **Age** | **Gender** | **Weight (kg)** | **Place of work** | **Type of work**  **(Task)** | **Duration**  **of work** | **Handle and contact chemical substance** | **Place of residence** | **Suffer from disease** | **Internal metallic supports** | **Physical activity** | **Fish consumption** | **Rice consumption** | **Type of drinking and cooking water** | **Dental amalgam** | **Smoking** | **Hair dyeing** |
| ICL_1 | 35 | M | 75 | Hg cell & HCl plant | Supervisor for 3 year | 6 years | Yes, 8 h/D | Faisalabad, near farms (Rural) | Yes | No | 3.5 h/w | 250 g/w  (Freshwater fish) | White rice  >250g/w | Well (ground) water | No | Yes | No |
| ICL_2 | 30 | M | 58 | Production (NaOH) | Supervisor | 13 years | Yes, 8 h/D | Lahore/  Shahdra (Urban) | No | No | Yes | No | Brown rice  250 g/w | Bottled water | No | No | Yes  Artificial |
| ICL_3 | 35 | M | 65 | Maintenance Fitter polyester | Fitting, maintenance of Hg cell | 7 years | Rarely | Shahdra Town  (Simi rural) | No | No | No | 250g/w Freshwater fish, 50g marine fish (rarely) | White  250 g/d  Brown rice 50 g/m | Tap Water | No | No | No |
| ICL_4 | 42 | M | 77 | Maintenance Sector | Maintenance of Hg cell | 21 years | Yes  12-14 h/D | Lahore, semi rural | Yes  10 yeas ago | No | Yes,  2 h/w | 250g (Freshwater fish) rarely | White rice  100 g/w | Tap Water | No | No | Yes  Artificial |
| ICL_5 | 55 | M | 72 | Cell room (fitter) | Maintenance | 20 years |  | Muridekay Fisal Colony, semi urban | No | No | Yes  8 h /w | 100g /w (Freshwater fish) | White rice  100 g/w | Tap Water | No | No | No |
| ICL_6 | 29 | M | 70 | NCR/SER | Maintenance, cell room | 10 years | Metal fume, NaOH vapor/  11-12 h/d | Lahore / Hekimpura  Near ICL | No | No | Yes  4 h /d | 150g /w (Freshwater fish) | White rice  250 - 750 g/w | Tap Water | Yes, 2 | No | No |
| ICL_7 | 38 | M | 56 | NCR/SER | Maintenance, cell room | 8 years | Metal fume, NaOH vapor/  11-12 h/d | Lahore / Hekimpura  Near ICL | Yes  12 yeas ago | No | Yes  8 h / d | 50g//w (Freshwater fish) | White 50 g/w  Brown rice 100 g/w | Tap Water | No | Yes | Yes  Artificial |
| ICL_8 | 54 | M | 54 | Maintenance Sector | Maintenance | 30 years | Exposure to Cl_2_ & Hg, 8 h/ d | Muridekay / Lahore / Canal Park near ICL | Yes | No | No | >250g/w  (Freshwater fish) | White and brown rice  300-900 g/w | Tap Water | No | No | No |
| ICL_9 | 50 | M | 68 | Rigger  (Maintenance) | Rigger | 10 years | Cl_2_, Hg,, NaOH,  8H /d | Lahore, near ICL plant | No | No | No | No | Whit rice  150 g/w | Tap Water | No | No | No |
| ICL_10 | 46 | M | 75 | Hg cell  (Maintenance) | Line gas leakage, Maintenance | 6 years | NaOH, Hg leakage (8h/d) | Kamonki, Raza Abad  Urban | No | No | No | > 250g/m  (Freshwater fish) | White rice  >250 g/w | Tap water | No | No | No |
| ICL_11 | 24 | M | 52 | SCR  (Hg /DSA) | Hg /DSA | 2 years | Hg fumes inhaled (8h/d) | Kala Shah Kaku/ Lahore / Rural /ICL | No | No | Some times | 200g/m  (Freshwater fish) | White rice 300 g/d | Tap water | No | No | Yes artificial |
| ICL_12 | 50 | M | 70 | Production department | Hg /DSA | 32 years | No | Baghbanpura/ Lahore semi urban | No | No | Yes  3h/d | 250g/m  (Freshwater fish) | White rice  100 g/m | Tap water | No | No | No |
| ICL_13 | 32 | M | 80 | Whole area | Hg /DSA | 4.5 years | NaOH and Cl_2_ | Rana / Lahore semi urban | No | No | Yes  3h/d | 50g/m  (Freshwater fish) | White rice  100 g/m | Tap water | No | No | Yes artificial |
| ICL_14 | 36 | M | 84 | Engineering department | Fitter | 7 yeas | Hg, acid, CL_2_ (8h/d) | Lahore near ICL | No | No | Yes  8 h/w | 200 g rarely  (Freshwater fish) | White rice  150 g/w  Brown rice  200 g/w | Tap water | Yes  2 | No | No |
| ICL_15 | 30 | M | 65 | Rigger work of folding | Fitter | 8 years | On Hg plant (8h/d) | Ferozwala /Lahore | No | No | Yes  3 h/w | No | White rice  100 g/w | Tap water | Yes  3 | No | No |
| ICL_16 | 26 | M | 58 | Fitters | Fitter | 7 years | Hg, NaOH, HCl (12h/d) | Rani complex /Lahore | Yes | No | No | No | White rice  100 to 300 g/w | Tap water | Yes  2 | No | No |
| ICL_17 | 48 | M | 72 | Managing | Production of plant | 11 years | -- | Kala Shah Kaku/ Lahore | No | No | Yes  8 h/d | 50 g rarely  (Freshwater fish) | White rice  100 g/w | Tap water | Yes  2 | No | Yes artificial |
| ICL_18 | 50 | M | 64 | Maintenance | Supervisor | 25 years | Cl_2_ sometimes 12h/d | Shahra / Lahore | No | No | Yes | 100 g /m  (Freshwater fish) | White rice 100 to 300 g/w | Tap water | No | No | Yes artificial |
| ICL_19 | 30 | M | 68 | Chemical sector | Maintenance | 1 year | Cl_2_ gas  12h/d | Lahore | No | No | Yes | 250 g/m  (Freshwater fish) | White 250g/w  Brown rice 200g/w | Tap and bottled water | No | No | No |
| ICL_20 | 25 | M | 52 | As a worker | Deal with Hg cell | 4 years | Hg and others (8h/d) | Kala Shah Kaku/ Lahore | No | No | No | No | White rice  250-750 g/w | Tap water | No | No | N0 |
| ICL_21 | 22 | M | 77 | Manual plant operator Hg | Production of plant | 3 years | Hg, leakage (8h/d) | Muridekay / Lahore / Canal Park | Yes | No | No | 250 g/m  (Freshwater fish) | No | Tap and  bottled water | No | No | Yes artificial |
| ICL_22 | 54 | M | 70 | Brine plant  Maintenance | Fitter | 15 years | Acid and Cl_2_ (12h/d) | Kala Shah Kaku/ Lahore | Yes | No | Yes  7 h/d | More than 250 g/m  (Freshwater fish) | White rice  100 g/w | Bottled H_2_O | No | Yes  5-10 h/d | No |
| ICL_23 | 25 | M | 25 | Maintenance | Assistant manager | 1 year | Hg  8h/d | Quetta city  urban | Typhoid  9 years ago | No | No | 250g rarely  (Freshwater fish) | White 200 g/w  Brown rice 250g/w | Well and tap water | Yes  1 | No | No |

| **Group No 2: SCL chloralkali workers** | | | | | | | | | | | | | | | | | |
| --- | --- | --- | --- | --- | --- | --- | --- | --- | --- | --- | --- | --- | --- | --- | --- | --- | --- |
| **Sample ID** | **Age** | **Gender** | **Weight (kg)** | **Place of work** | **Type of work**  **(Task)** | **Duration**  **of work** | **Handle and contact chemical substance** | **Place of residence** | **Suffer from disease** | **Internal metallic support** | **Physical activity** | **Fish consumption** | **Rice consumption** | **Type of drinking and cooking water** | **Dental amalgam** | **smoking** | **hair dyeing** |
| SCL_1* |  |  |  |  |  |  |  |  |  |  |  |  |  |  |  |  |  |
| SCL_2 | 60 | M | 75 | Production area | Senior Manager | 20 year | Cl_2_ & HCl  8h/d | Gulshan Ravi, Lahore | Yes | No | Yes  3 to 4h/d | 150g rarely  (Freshwater) | White rice  100 g/w | Tap water | No | No | No |
| SCL_3 | 45 | M | 72 | Production area | Senior foreman | 25 year | NaOH, 8h/d | Faisalabad, Chak | No | No | No | No | White rice  (50 g/w) rarely | Tap water | Yes  2 | No | Yes  Artificial |
| SCL_4 | 51 | M | 63 | Mechanical section | Assistant foreman | 5 year | NaOH, HCl, 8h/d | Faisalabad, Chak | No | No | Yes  11 h/d | No | White  150 g/d  Brown rice  150 g/d | Tap water | No | No | No |
| SCL_5 | 35 | M | 80 | Mechanical section | Chemical handling | 12 year | NaOH, HCl & NaOH  8 h/d | Faisalabad, Chak | No | No | Yes  4 h/d | No | White and Brown rice  200 g/d | Tap water | No | No | No |
| SCL_6 | 40 | M | 55 | Assistant Foreman | Chemical handling | 18 year | NaOH,  8 h/d | Faisalabad | No | No | Yes  4 h/d | No | White and Brown rice  150 g/d | Bottled water | No | No | No |
| SCL_7 | 47 | M | 68 | Mechanical Dept. | Senior foreman | 26 year | Cl_2_, HCl, NaOH, 10h/d | Faisalabad, Chak | No | No | Yes  5 h/d | 150g  (Freshwater) | White rice  200 g/m | Bottled water | No | No | Yes  Artificial |
| SCL_8 | 56 | M | 82 | Manager | To look after system | 12 year | Yes  8-10h/d | Shah Kot / Faisalabad | Yes | No | Yes  4 h/d | 200 g rarely  (Freshwater) | White rice  200 g/w | Water well  (Ground water) | No | No | Yes  Artificial |
| SCL_9 | 50 | M | 70 | Rigging | Process and production | 24 year | Yes  8 h/d | Shah Kot / Nankana sahab | No | No | No | 50g /m  (Freshwater) | White rice  200 g/w | Tap water | No | No | Yes  Artificial |
| SCL_10 | 24 | M | 68 | Process Dept. | Process and production of NaOH | 9 years | Chlorine gas  8 h/d | Shah Kot / Nankana sahab | No | No | No | No | White rice  200 g/w | Tap water | No | No | Yes  Artificial |

* Data not available

| **Group No 3: Control group 1 (general public)** | | | | | | | | | | | | | | | | | |
| --- | --- | --- | --- | --- | --- | --- | --- | --- | --- | --- | --- | --- | --- | --- | --- | --- | --- |
| **Sample ID** | **Age** | **Gender** | **Weight (kg)** | **Place of work** | **Type of work**  **(Task)** | **Duration**  **of work** | **Handle and contact chemical substance** | **Place of residence** | **Suffer from disease** | **Internal metallic support** | **Physical activity** | **Fish consumption** | **Rice consumption** | **Type of drinking and cooking water** | **Dental amalgam**  **(Hg dental filling)** | **Smoking** | **Hair dyeing** |
| C1_1 | 20 | M | 68 | Student | Surgeon | --- | Amalgam | Lahore | No | No | Yes  15 h/d | 250 g (rarely) | White rice 150g/w | Tap water | No | No |  |
| C1_2 | 40 | M | 60 | Punjab University | Gardening | 10 years | No | Lahore | No | No | No | 200 g/m  (Freshwater fish) | White rice  250 g/d | Tap water | Yes  2 | No |  |
| C1_3 | 25 | M | 70 | Bank | Manager | 4 years | No | Lahore | No | No | Yes  1-2 h/d | 200 g/m | White rice  250 g/d | Tap and  bottled water | Yes 2 | No |  |
| C1_4 | 27 | M | 79 | College | Teacher | 5 years | No | Lahore | No | No | No | 150 g/m | White rice  150 g/w | Tap water | No | No |  |
| C1_5 | 32 | M | 60 | Health | Teacher | 5 years | No | Lahore | No | No | Yes  48 h/w | No | White rice  150 g/w | Tap water | No | Yes |  |
| C1_6 | 16 | M | 44 | University | Student | --- | No | Lahore | No | No | Yes  12 h/d | No | White rice  150 g/w | Tap water | Yes  1 | No |  |
| C1_7 | 45 | F | 73 | Vocational instructor | Attendant | 5 years | Yes | Lahore | No | No | No | 100g rarely | White rice  200 g/w | Tap and  bottled water | Yes  1 | No |  |
| C1_8 | 69 | F | 65 | --- | Teacher | --- | No | Lahore | No | No | No | 200g  (rarely) | White rice  250 g/d | Tap water | Yes 2 | No |  |
| C1_9 | 20 | M | 79 | University | Student | --- | No | Lahore | No | No | Yes  2-3 h/d | 200 g/m | White and brown rice  250 g/w | Tap water | No | No |  |
| C1_10 | 19 | M | 58 | Shopkeeper | Shopkeeper | 3-4 years | No | Lahore | No | No | No | No | White  150 g/w | Tap water | No | No |  |
| C1_11 | 16 | M | 47 | University | Student | --- | No | Lahore | Yes | No | No | No | White rice  150 g/w | Tap water | No | No |  |
| C1_12 | 19 | M | 60 | University | Student | --- | No | Lahore | No | No | Yes  10 h/d | 150 g/m | White and brown rice  100 g/w | Tap and  bottled water | No | No |  |
| C1_13 | 37 | M | 65 | University | Secretary officer | 6 years | No | Lahore | No | No | No | 200 g/m | White rice  200 g/m | Tap water | Yes  1 | No |  |
| C1_14 | 23 | M | 64 | University | Student | --- | No | Lahore | No | No | Yes  7 h/d | 250 g  (rarely) | White rice  200 g/w | Tap water | No | No |  |
| C1_15 | 20 | M | 66 | University | Student | --- | No | Lahore | No | No | Yes  9 h/d | No | White rice  250 g/w | Tap water | No | Yes |  |
| C1_16 | 18 | M | 65 | University | Student | --- | No | Lahore | No | No | Yes  14 h/d | 250 g  Rarely | White rice  100 g/w | Tap and  bottled water | Yes  2 | No |  |
| C1_17 | 22 | M | 78 | University | Student | --- | No | Lahore | Yes | No | Yes 7h/d | 250 g  (rarely) | White and brown ride  250 g/w | Tap water | No | Yes |  |
| C1_18 | 25 | M | 90 | University | Student | --- | No | Lahore | No | No | Yes 18h/d | 250 g/m | White rice  150 g/d | Tap water | No | No |  |

| **Group No 4: Control group No (people live in the vicinity of ICL chloralkali plant)** | | | | | | | | | | | | | | | | | |
| --- | --- | --- | --- | --- | --- | --- | --- | --- | --- | --- | --- | --- | --- | --- | --- | --- | --- |
| **Sample ID** | **Age** | **Gender** | **Weight (kg)** | **Place of work** | **Type of work**  **(Task)** | **Duration**  **of work** | **Handle and contact chemical substance** | **Place of residence** | **Suffer from disease** | **Internal metallic support** | **Physical activity** | **Fish consumption** | **Rice consumption** | **Type of drinking and cooking water** | **Dental amalgam**  **(Hg dental filling)** | **Smoking** | **Hair dyeing** |
| C2_1 | 46 | M | 45 | Gardner | Gardening | 12 | No | Kalashah kaku, near ICL | No | No | No | No | White and brown rice  200 g/w | Well and tap water | No | No | No |
| C2_2 | 42 | M | 110 | Security sector | Security guard | 15 | chlorine, HCl | Kalashah kaku, near ICL | No | No | No | 250 g rarely  (Freshwater fish) | White rice 150 g/w  Brown rice >250 g/w | Tap water | No | No | Yes  Natural |
| C2_3 | 32 | M | 72 | Refrigeration | Mechanic | 17 | chlorine, HCl | Kalashah kaku, near ICL | No | No | Yes  4 h/w | >250 g rarely  (Freshwater fish) | White and  brown rice >250 g/w | Tap water | Yes  1 | No | Yes  Natural |
| C2_4 | 30 | M | 50 | Chemical factory | Fair price shop helper | 5 | mercury fumes | Kalashah kaku, near ICL | No | No | No | No | Brown  200 g/w | Well water | No | Yes | Yes artificial |
| C2_5* | 33 | M | 74 | Telephone department | Telephone technician | 13 | chlorine, HCl | Kalashah kaku, near ICL | No | No | Yes  20 h/d | No | White and brown rice  >250 g/w | Well and tap water | No | No | No |

* Hair samples were not provided

**Supplementary Materials and Methods**

**Chemicals and reagents**

All chemicals used were of analytical trace select grade unless stated otherwise. 70% HNO_3_ (AnalR grade; VWR, Lutterworth, UK) and 25% *w/w* aqueous tetramethylammonium hydroxide (99.9999 % (metal basis); Alfa Aesear, Heysham, UK) were used for sample digestion and solubilisation, respectively. Acetate-acetic acid buffer (0.5 M) was prepared with acetic acid (100 %, HiPerSolv® Chromanorm; VWR, Lutterworth, UK) and the pH was adjusted with NaOH (laboratory reagent grade; Fluka Analytical, Gillingham, UK). Mercury species were propylated with sodium tetrapropyleborate (Chemos, Germany) and extracted into 2,2,4-trimethylpentane (Chromasolv® Plus, for HPLC, ≥ 99.5%; Sigma-Aldrich, Gillingham, UK). Me^201^Hg^+^ for Hg speciation was prepared from ^201^HgO enriched standard solution (Oak Ridge National Laboratory, Oak Ridge, TN, USA) following the literature.(Rodriguez Martin-Doimeadios et al. 2002) Solution of enriched ^199^HgO was used for inorganic Hg quantification (Oak Ridge National Laboratory, Oak Ridge, TN, USA). For the CV-AFS analysis a solution of 2% (m/v) tin(II) chloride (purchased as tin(II) chloride dehydrate (98%), Alfa Aesear, Heysham, UK) and 1.2 M HCl (AnalR grade; VWR, Lutterworth, UK) in double distilled water was used as a reductant.

A stock solution of inorganic Hg standard and thallium (Tl) standard (M21, TraceCERT®; Sigma-Aldrich, Gillingham, UK) were used for preparation of calibration standards and as internal standard, respectively during ICP-MS analysis. Double distilled water was purified using Aquatron A4,000D (Bibby Scientific Ltd, Stone, UK). The certified reference materials (CRM) used were IAEA-085 (human hair) from International Atomic Energy Agency (Vienna, Austria) and NIES-No. 13 (human hair) from National Institute for Environmental Studies (Tsukuba, Japan).

**References**

Rodriguez Martin-Doimeadios RC, Stoichev T, Krupp E, Amouroux D, Holeman M, Donard OFX. 2002. Working methods paper: Micro-scale preparation and characterization of isotopically enriched monomethylmercury. Applied Organometallic Chemistry 16:610-615.
